# Passive drift or active swimming in marine organisms?

**DOI:** 10.1098/rspb.2016.1689

**Published:** 2016-12-14

**Authors:** Nathan F. Putman, Rick Lumpkin, Alexander E. Sacco, Katherine L. Mansfield

**Affiliations:** 1 Cooperative Institute for Marine and Atmospheric Studies, Rosenstiel School of Marine and Atmospheric Science, University of Miami, Miami, FL 33149, USA; 2 Atlantic Oceanographic and Meteorological Laboratory, National Oceanic and Atmospheric Administration, Miami, FL 33149, USA; 3 Department of Biology, University of Central Florida, Orlando, FL 32816, USA

**Keywords:** ocean currents, swimming, passive drift, ocean circulation model, satellite telemetry, sea turtle

## Abstract

Predictions of organismal movements in a fluid require knowing the fluid's velocity and potential contributions of the organism's behaviour (e.g. swimming or flying). While theoretical aspects of this work are reasonably well-developed, field-based validation is challenging. A much-needed study recently published by Briscoe and colleagues in *Proceedings of the Royal Society B* compared movements and distribution of satellite-tracked juvenile sea turtles to virtual particles released in a data-assimilating hindcast ocean circulation model. Substantial differences observed between turtles and particles were considered evidence for an important role of active swimming by turtles. However, the experimental design implicitly assumed that transport predictions were insensitive to (i) start location, (ii) tracking duration, (iii) depth, and (iv) physical processes not depicted in the model. Here, we show that the magnitude of variation in physical parameters between turtles and virtual particles can profoundly alter transport predictions, potentially sufficient to explain the reported differences without evoking swimming behaviour. We present a more robust method to derive the environmental contributions to individual movements, but caution that resolving the ocean velocities experienced by individual organisms remains a problem for assessing the role of behaviour in organismal movements and population distributions.

## 1. Introduction

Understanding the mechanisms driving organismal movement has long-been viewed as an essential component to the conservation and management of species and ecosystems [1,2]. For marine animals, a topic of considerable importance is how to assess the extent to which organisms can influence their movements and population distribution within the dynamic ocean [2–4]. Early on, ocean currents were presumed to dominate organisms’ movements, owing to limited swimming capacity relative to ocean velocity and/or a limited ability of animals to direct their swimming in the barren sensorial-environment of the open sea [2]. Over the years, the validity of both of these assumptions has been eroded [5,6] and there is general consensus that directed swimming, even at seemingly trivial speeds, could have profound consequences for the movements, fitness, and distribution of marine organisms [7–9]. Even so, the difficulty of performing experiments in the open ocean has meant that very few direct tests to compare passive drift and active swimming have been performed [10–12].

A much needed field-based study in this area was recently reported by Briscoe *et al*. [13]. They used satellite telemetry to examine the movements of juvenile sea turtles, a taxa at the centre of a decades-old controversy as to whether they behave as ‘passive migrants’ in their early years during which transoceanic movements occur [14,15]. This study comes from a distinguished team that pioneered field-based approaches to track the movements of juvenile turtles in the open ocean [16–19] and their sustained contributions [20–26] have yielded invaluable insight into what had been considered ‘the lost years’ of sea turtles [14]. More recently, Briscoe *et al*. [13] compared the movements and distribution of captive-reared loggerhead sea turtles (*Caretta caretta*), released off the coast of Japan, to ocean currents from a global ocean circulation model. They concluded that turtle velocities and distribution could not be the result of ocean currents alone, indicating the importance of directional swimming during the oceanic migration of juveniles. Implicit in their analyses were the assumptions that transport predictions are insensitive to (i) start location, (ii) tracking duration, (iii) depth, and (iv) physical processes not depicted in the ocean circulation model.

Here, we use a combination of modelling and *in situ* data to demonstrate that the magnitude that each of the above parameters varied within the study by Briscoe *et al*. [13] can substantially influence transport predictions. While it might ultimately prove true that Japanese loggerhead turtles engage in ‘active dispersal’, our analyses reveal that the methods employed by Briscoe *et al*. [13] fail to show this with any certainty. We conclude with suggestions for more robust investigation of the tracking data and highlight important elements of experimental design for consideration in future studies.

## 2. Material and methods

### (a) Background

Briscoe *et al*. [13] obtained data on turtle movement by laboratory-rearing 44 loggerhead sea turtles to an age of 1–3 years (29.7–37.5 cm straight carapace length), outfitting the turtles with satellite transmitters and releasing them on two separate days, 9 April 2010 (*n* = 17) and 12 July 2011 (*n* = 27). The reported locations of release differ between their methods section and electronic supplementary material. Their methods imply turtles were released at a single point on each of those days (29° N, 130° W in April 2010 and 36° N, 141° E in July 2011), whereas their electronic supplementary material indicates that the first release was a single point (29.7° N, 130.5° W), but the second release included more than a dozen locations spanning latitudes 34.8° N to 37.5° N and longitudes 141.2° W to 146.6° W. Tracking data were obtained for turtles through ARGOS-CLS and filtered by a Bayesian state-space switching model [27] to estimate each turtles' location at 24 h intervals. Track durations ranged from 173 days to 865 days (mean = approx. 469 days) post-release.

**Figure 1.**
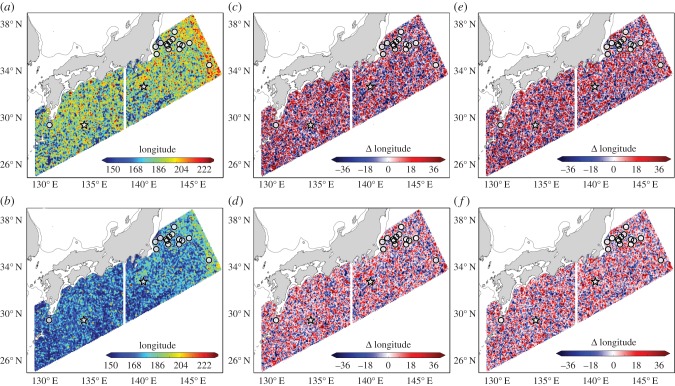
The influence of release location, date, and tracking duration on predicted eastward transport in the surface layer of Global HYCOM. (*a*) Particles were tracked for 865 days, the longest track duration in Briscoe *et al*. [13]. (*b*) Particles were tracked for 469 days, the mean track duration. (*a*,*b*) The maximum eastward longitude travelled by a particle released in a given location for the turtle release dates (9 April 2010, southwest of the white dividing line; 12 July 2011, northeast of the white dividing line). (*c*,*d*) The difference between the maximum eastward longitude at a given location for the turtle release date and the maximum eastward longitude at that same location the preceding day. (*e*,*f*) The difference between the maximum eastward longitude at a given location for the turtle release date and the maximum eastward longitude at that same location the following day. In all panels, circles indicate the release sites of turtles, stars are the approximate position of particle release, inferred from the supplementary information and from figure 1 of Briscoe *et al*. [13], respectively. The thin black line delineates the continental shelf (200 metre isobath). Predictions of transport vary considerably over distances of a few km and within 24 h periods. Not taking into account this variability will almost certainly obfuscate the ability to assess the role of ocean currents on organismal movements.

To assess whether ocean currents accounted for the turtles' movements, Briscoe *et al*. [13] released virtual particles at two locations in the surface layer of Global Hybrid Coordinate Ocean Model (HYCOM) output [28] using the particle-tracking software Ichthyop v. 3.2 [29]. Global HYCOM hindcasts are daily snapshots of ocean velocity at a spatial resolution of 0.08°. HYCOM is an eddy-resolving model that assimilates *in situ* and satellite observations to depict oceanic conditions that occurred at specific times in the past [28]. The location of particle releases were not reported, but they state that ‘… particles were released approximately 50 km offshore within a zone that corresponded to the main deployment locations for 2010 and 2011’ [13]. Figure 1 of their paper implies the particle release was substantially eastward of the 9 April 2010 turtle release and southward of the 12 July 2011 turtle release(s). Particles were released at these sites the day of the release as well as the day before and after (1 000 particles each day). Particle trajectories were computed for 865 days, corresponding to the longest track duration for the turtles. The speed, direction, and distribution of turtles and particles were then compared by longitude. (A second simulation was also performed, in which particle trajectories were computed for 4 years, to test whether transpacific transport was possible and was not used in direct comparison with the tracking data for the turtles.)

To determine how much of an animal's movement is due to the ocean currents it encounters, one must have ocean current information coincident with the animal's location [2–4]. It may be problematic, therefore, that Briscoe *et al*. [13] released virtual particles at locations and times different from release sites of turtles, compared the distribution of turtles and particles tracked for different durations, and compared velocities (speed and direction of movement) of turtles and particles that were not spatially or temporally concomitant. Similarly, particles tracked at 0 m in Global HYCOM might not correspond to the same depth at which turtles travelled, as there is no mention of turtle diving (though other studies suggest this life-stage spends considerable time between 15 m and the ocean surface (e.g. [12,18,21])). Moreover, they implicitly assumed that physical processes not resolved in Global HYCOM output would result in a negligible contribution to differences between turtles and particles, as they did not test the sensitivity of their approach to depict actual oceanic conditions. In sum, we cannot know whether turtle and particle tracks differ because of the behaviour of turtles, because different oceanic conditions were experienced by turtles and particles, or because of error in the ocean circulation model. Below, we examine how such factors could explain the differences reported between turtle movement and modelled ocean currents.

### (b) Influence of start location on transport predictions

To test the influence of release location on transport predictions, we performed particle tracking simulations with Ichthyop v. 2 particle tracking software [29] and the surface layer of Global HYCOM [28]. We defined a release zone seaward of the 200 m isobath between latitudes 25° N and 39° N and longitudes 129° E and 148° E. Within this region, 10 000 virtual particles were released from random locations west of 137.5° E on 9 April 2010. To the east of 137.5° E, 10 000 virtual particles were released from random locations on 12 July 2011. These regions and dates correspond to the release sites of turtles in Briscoe *et al*. [13]. Trajectories were computed at 30 min intervals using the Runge–Kutta fourth-order time-stepping method in Ichthyop v. 2 particle tracking software [29]. From each start location, the maximum eastward longitude was determined for 865 days (the maximum turtle track duration and the duration of particle transport chosen by Briscoe *et al*. [13]). We used the cubic interpolation function in SciPy (scipy.org) to create a uniform surface at the resolution of Global HYCOM (0.08° latitude × 0.08° longitude) to show maximum eastward transport by start location.

### (c) Influence of track duration on transport predictions

To test the influence of track duration on transport predictions, we performed the same analyses described in §2b, but computed the maximum eastward longitude of each particle's trajectory after 469 days, the mean turtle track duration in Briscoe *et al*. [13].

### (d) Influence of start date on transport predictions

Briscoe *et al*. [13] released particles into Global HYCOM the day before and after the turtle release data. To determine what influence these different start dates might have on transport predictions, we performed the same analyses described in §2b and c, but for the day before and after the turtle release date (days 8 and 10 April 2010, and 11 and 13 July 2011). We then computed the maximum eastward transport by start location for both 865 and 469 days. To determine the difference in transport predictions between releases differing by 24 h, we subtracted the maximum eastward longitude surface estimated for dates of turtle release (9 April 2010/12 July 2011) from the maximum eastward longitude surfaces obtained the day before and the day after.

### (e) Influence of depth on transport predictions

We tested whether variation in transport predictions occurred at the range of depths that are most often encountered by oceanic-stage juvenile sea turtles, the surface down to 15 m [12,18,21]. We opted to use data from the National Oceanographic and Atmospheric Administration (NOAA) Global Drifter Programme in these analyses, as Global HYCOM does not depict all of the physical processes that may contribute to an object's movement at the ocean surface (e.g. waves and direct forcing by winds). Drifters are deployed with drogues (i.e. sea anchors) centred at 15 m depth and are equipped with sensors that relay whether the drogue is attached or has been lost. The movement of drogued drifters is dominated by currents in the upper 15 m of the water column, whereas undrogued drifter movements are the result of currents and near-surface processes (windage, Stokes drift, etc.). For these analyses, two transition matrices were computed from the 36 years of drifter data, one matrix for drogued drifters and one matrix for undrogued drifters. This was done as follows [30–32]: the world was divided into an array of regular 1° × 1° bins, and all drogued or undrogued drifters in the historical dataset from 1979 to 2015 were identified that passed through each bin. The locations of these drifters were then found 30 days later, which is many times the autocorrelation timescale of drifter motion [33]. These locations were used to calculate a transition matrix **P***_ij_* that contains the odds for each bin *j* that a drifter will occupy it given that the drifter was in bin *i* 30 days before. Because some drifters will die due to technical reasons or because they were picked up or ran aground, the values in **P***_ij_* are rescaled so that, summed over *j*, the total odds are 1 for all values of *i* [31]. Computations were made from the start locations reported in Supplemental table S1 of Briscoe *et al*. [13]. Drifter movements among 1° × 1° bins were computed at 30 day intervals for the corresponding track durations (rounded to the closest 30 day period, e.g. the turtle tracked for 865 days corresponded to an 870 days drifter simulation).

This approach with drifters was used because it was more important to fully characterize the physical processes potentially contributing to differences in velocity over the range of depths juvenile loggerheads frequent, rather than characterize oceanographic conditions that corresponded to particular temporal periods. However, because the statistical nature of this approach ignores potentially unique, seasonal or annual variability in ocean currents, the transition-matrix is not well suited to assess the ocean velocities encountered by specific animals at particular places and times. Therefore, to provide further context to the Briscoe *et al*. [13] study, we performed similar analyses using the surface layer of Global HYCOM [28] during the study period. We used Ichthyop v. 2 software [29] to release 1 000 virtual particles at the same deployment locations and dates as the 44 turtles in Supplemental table 1 of Briscoe *et al*. [13]. Particle trajectories were computed at 30 min intervals using the Runge–Kutta fourth-order time-stepping method for the track duration for each turtle. A daily location was recorded for each particle and the number of particles within each 1° × 1° grid cell was summed across each day of the simulation. To compare the distribution predictions of the drifter matrices to the virtual particle approach, the drifter surfaces were multiplied by 200 012 937 (the number of daily positions in the virtual particle simulation).

### (f) Influence of physical processes not resolved in ocean circulation models on transport predictions

As alluded to above, a number of physical processes that contribute to the velocity of objects in the ocean are not well-represented in most global ocean circulation models, including Global HYCOM. These include factors such as Stokes drift (wave-induced velocity), tides, direct forcing by winds (windage), as well as all processes occurring at spatial and temporal scales finer than the ocean circulation model's resolution [4]. In order to assess whether swimming behaviour is responsible for differences between observed distributions of organisms and those predicted from an ocean circulation model, it is prudent to determine to what extent differences in results might be attributable to model error. Treating the tracking data from a drifter as if it were the movement of an animal provides a valuable ‘control’ for field-based experiments [4,12].

As an example, we tested the sensitivity of various methods to infer swimming behaviour in marine animals by comparing predictions of ocean velocity from Global HYCOM to a drifter trajectory (ID 35228) from the Global Drifter Array that coincided with the region of turtle release in [13]. For simplicity of presentation and to make results most relevant for comparison with Briscoe *et al*. [13], we performed analyses using the surface layer of Global HYCOM. The drogue on this drifter was attached for the first 230 days of its life, corresponding to the period analysed here. Tracking data were examined from 18 June 2011 (37.044° N, 132.458° W) through 2 February 2012 (37.276° N, 152.730° W) at daily intervals (00.00 h GMT). Briscoe *et al*. [13] compared the track velocities of turtles with the velocities of virtual particles released at a site distant to that of the turtle release sites. We tested the ability of the approach to characterize the velocity of a passively drifting object by releasing 500 virtual particles in a 0.08° × 0.08° rectangle that was 0.5° in latitude to the south of the drifter's start position. Particles were tracked for 230 days, the duration of the drifter track. A similar analysis was performed in which 500 virtual particles were released in a 0.08° × 0.08° rectangle centred on the drifter's initial position and tracked for 230 days. Track and mean particle velocity (speed and direction) were compared at daily intervals. We also released 200 virtual particles in 0.08° × 0.08° rectangles centred on the daily locations of the drifter and tracked each particle for 1 day. In this case, the particle closest to the subsequent drifter location was used to compare particle speed with track speed. We note that this comparison intentionally favours a model/observation match if possible, as opposed to using the centre of mass of the simulated particle cloud. In each of the above cases, particle trajectories were computed at 30 min intervals using the Runge–Kutta fourth-order time-stepping method and recorded daily.

We assessed whether track velocity and the three approaches for inferring velocity from Global HYCOM were equivalent by comparing the daily speed estimates with a Kruskall–Wallis test and daily direction estimates with a Mardia–Watson–Wheeler test. We then assessed whether particle speeds were correlated with the speed of the drifter using Spearman's rank-order correlation and whether particle directions were correlated with the direction of the drifter using Circular–Circular correlations.

## 3. Results

### (a) Influence of start location on transport predictions

Tracking virtual particles offshore of Japan shows the potential for substantial variation in transport (figure 1*a*). Particles released on the same day at locations separated by only tens of a kilometre might travel no further east than 150° E or past longitude 150° W, a more than 4 000 km difference over the course of the 865 day tracking period. The spatial heterogeneity in maximum eastward transport (figure 1*a*) highlights the importance of co-localizing measures of ocean currents with tracking data. It is conceivable that differences in distribution between particles and turtles in Briscoe *et al*. [13] are the result of differences between particle release sites and sites of turtle deployments.

### (b) Influence of track duration on transport predictions

Eastward transport was significantly reduced for particles tracked for 469 days (figure 1*d*), compared with those tracked for 865 days (figure 1*a*). Only 19% of particles were predicted to cross into the Western Hemisphere after 469 days (figure 1*b*), whereas 64% of particles did when allowed to drift for 865 days (figure 1*a*). Thus, in the context of assessing the role of ocean currents on broad-scale distributions of marine turtles, it should be expected that particles drifting for more than a year longer than the ‘average’ turtles in Briscoe *et al*. [13] would travel further east, even if turtles were entirely passive (figure 1*a*,*b*).

### (c) Influence of start date on transport predictions

Predictions of maximum eastward transport for particles released from the same locations but a day apart indicate that an increase or decrease of eastward movement by 30° longitude is possible over a 469 or 865 day tracking period (figure 1*c*–*f*). Thus, ocean currents at a location even as little as 24 h apart from when an organism was in that same area might be entirely unrepresentative of the oceanic conditions that were experienced. In the context of Briscoe *et al*. [13], releasing particles over multiple days would result in predicting a broader range of transport possibilities than were available to turtles, likely increasing the chances of detecting statistical differences between turtles and particles.

### (d) Influence of depth on transport predictions

**Figure 2.**
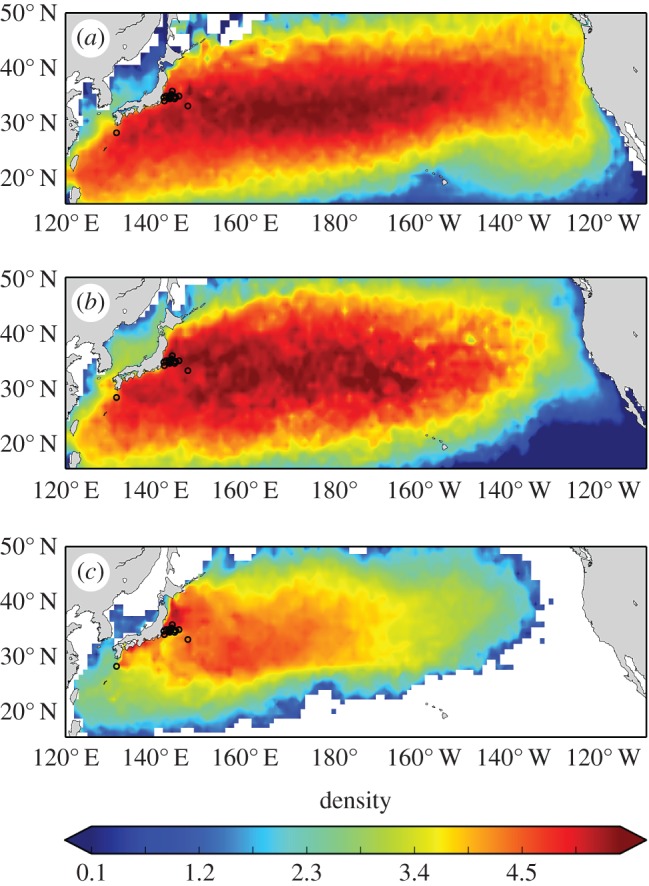
Predicted distributions assuming passive drift of (*a*) undrogued surface drifters, (*b*) drifters with drogues centred at 15 m, and (*c*) virtual particles released in the surface layer of HYCOM. Computations were made from the 44 start locations (black circles) and integrated through time for the corresponding track durations reported in Supplemental table 1 of Briscoe *et al*. [13]. Colours in each plot are log_10_-scaled relative to the HYCOM simulations, assuming a release of 1 000 virtual particles per release site, summed daily throughout the simulation. Panels (*a*,*b*) represent predictions taking into account 36 years of *in situ* oceanic conditions, whereas panel (*c*) depicts modelled conditions that occurred during the tracking experiment by Briscoe *et al*. [13].

The predicted distribution of undrogued drifters at the ocean surface was substantially eastward of the predicted distribution of drifters drogued to follow water movements at 15 m depth (figure 2*a*,*b*). The distribution of particles tracked within the surface layer of Global HYCOM (figure 2*c*) corresponded better to the predicted distribution of drifters drogued at 15 m depth (figure 2*b*). Less dispersion in the HYCOM predictions can be primarily attributed to fewer potential movement pathways realized under the unique release conditions in HYCOM (i.e. those corresponding to specific dates) compared with those summed over the 36 years of drifter data. The extent that predictions of distribution based on undrogued drifters, drogued drifters, and particles within the surface layer of HYCOM (despite co-localization of release sites and equivalent periods of drift) further highlights that physical processes alone can be responsible for divergence between model predictions and tracking data (figure 2).

Likewise, these results imply that the position of organisms in the water column could have considerable importance in determining the population's distribution. In the context of Briscoe *et al*. [13], it appears that if turtles remained at the ocean surface for extended periods, eastward transport would be enhanced, but spending a greater portion of time at depth would increase retention in the western Pacific. Briscoe *et al*. [13] proposed that the more westward distribution of turtles relative to virtual particles tracked at the ocean surface was the result of oriented swimming; an alternative explanation is that time at depth slowed the eastward progress of turtles (figure 2). In other words, vertical, rather than lateral, swimming may play an important role in the observed distribution of the turtles.

### (e) Influence of physical processes not resolved in ocean circulation models on transport predictions

**Figure 3.**
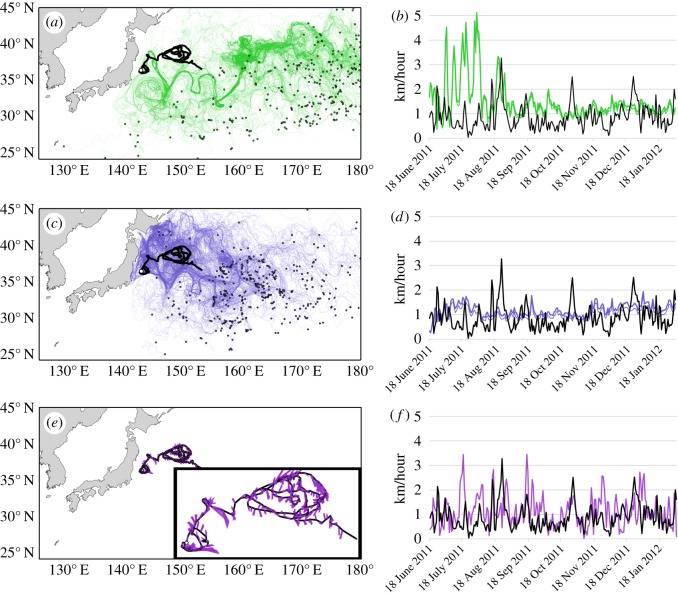
Tracks of virtual particles released within the surface layer of Global HYCOM relative to the tracks of a surface drifting buoy. (*a*) The thick black line shows the 230 days track of a drogued drifter ID 35 228 (start location indicated by the white circle). Green lines show the tracks of 500 virtual particles released 0.5° to the south of the drifter's start location (similar to Briscoe *et al*. [13]) and tracked for 230 days. Small dots indicate the final locations of virtual particles. (*b*) Graph showing daily drifter speed (black line) and the mean (±95% CI) speed of the 500 virtual particles at daily intervals in kilometres per hour. (*c*) Same as in (*a*), but showing tracks of 500 virtual particles released at the start location of the drifter (pale blue). (*d*) Same as in (*b*), but blue lines indicate the daily mean (±95% CI) speed of the 500 virtual particles released coincident with the drifter's start location. (*e*) Purple lines show the 1 day tracks of 200 virtual particles released on the daily positions of the drifter track. The inset surrounded by thick black lines shows a magnified view of the virtual particle trajectories relative to the drifter track. (*f*) Same as in (*b*,*d*), but showing the daily speed of the particle nearest to the subsequent location of the drifter (purple line) along the duration of the drifter track.

Daily measures of ocean current velocity significantly differed as assessed by drifter ID 35228, particles released 0.5° to the south of the drifter's start location and integrated for 230 days (figure 3*a*,*b*), particles released at the start location of the drifter and integrated for 230 days (figure 3*c*,*d*), and virtual particles released sequentially along the drifter track and integrated for 1 day (figure 3*e*,*f*) (speed: Kruskal–Wallis *H* = 139, *p* < 5 × 10^−30^, *n* = 230, d.f. = 3; direction: Mardia–Watson–Wheeler *W* = 98, *p* = 0, *n* = 230, d.f. = 3). A significant correlation was detected between daily drifter speed and the speeds of particles sequentially released along the track (Spearman's *r* = 0.235, *p* < 0.001, *n* = 230; figure 3*e*,*f*). Likewise, a significant correlation was found between daily drifter direction and the directions of particles released along the track (Circular–Circular correlation *r* = 0.299, *p* < 0.001, *n* = 230). By contrast, releasing particles at the start location of the drifter (figure 3*c*,*d*) or some distance away (figure 3*a*,*b*) resulted in ocean velocity estimates unrelated to drifter speed (Spearman's *r* = −0.002, *p* = 0.978, *n* = 230; Spearman's *r* = −0.096, *p* = 0.149, *n* = 230, respectively) or drifter direction (Circular–Circular correlation *r* = −0.06, *p* = 0.365, *n* = 230, Circular–Circular correlation *r* = −0.023, *p* = 0.729, *n* = 230, respectively).

These analyses indicate that care should be exercised when choosing an analytical approach to infer behaviour from tracking data, as approaches for quantifying ocean currents are not equivalent. Sequentially releasing particles along a track is clearly more meaningful for assessing ocean velocities relevant to organismal movements (figure 3*e*) compared with a single release of particles (figure 3*a*,*c*). However, even this more accurate approach is far from perfect. Subtracting particle velocity from track velocity, as is commonly done to derive the swimming velocity of an organism [4], would identify significantly oriented northward swimming by the drifter (median heading = 17°, Rayleigh's *r* = 0.143, *p* = 0.009, *n* = 230) at a median speed of 0.94 km h^−1^. The ability to fully resolve ocean currents remains a problem in assessing the role of swimming behaviour on organismal movements. Deviations between an animal's track and modelled currents cannot immediately be assumed to result from swimming behaviour; rather a combination of analyses and *in situ* observations are required to infer the relative contributions of drift and swimming to an organism's movement.

## 3. Discussion

Our analyses indicate that oceanic transport predictions are strongly influenced by location, date, tracking duration, and depth (figures 1 and 2). Furthermore, physical processes not characterized in ocean circulation models can result in substantial departures between predictions and the actual movements of a passive object in the ocean (figure 3). It is important to note that our analyses were chosen as simple demonstrations of how variability in ocean currents cause challenges for inferring the role of behaviour on organismal movements and distributions. For instance, considering ocean velocity at relevant depths is often more complex than determining whether the organism is at the surface or at 15 m depth (figure 2). The depths that turtles frequent likely vary among oceanic areas [34] and the impacts from winds (such as storms) may be more pronounced in certain areas and times [35]. Other complications include the possibility that young turtles might sporadically exhibit directional swimming, but otherwise drift [36]. As such, it is imperative that telemetry studies employ an experimental design that controls for such possibilities. Regardless, our results indicate that swimming behaviour should not immediately be assumed as the explanation for systematic differences between the movements and distributions of marine organisms and predictions based on ocean circulation models (figure 3) or, necessarily, estimates derived from *in situ* measurements of ocean currents (figure 2*a*,*b*).

Interestingly, the distribution of turtles in figure 1*a* of Briscoe *et al*. [13], appears to be well accounted for by the predicted distributions based on drogued drifters and particles tracked within the surface layer of Global HYCOM (figure 2*b*,*c*). This suggests that the discordance in large-scale distribution between modelled currents and turtle tracks reported by Briscoe *et al*. [13] could be due to the spatio-temporal mismatch of turtle and particle release sites and the longer tracking durations for particles relative to turtles. The stated reason for the spatial mismatch between turtle and particle release locations was ‘… to minimize the influence of coastal transport and retention unable to be quantified by HYCOM …’. However, turtle release locations were all seaward of the continental shelf (200 m isobath; figure 1) and thus ocean currents at those sites should be depicted reasonably well by Global HYCOM. The explanation for the temporal mismatch was so that particles would ‘experience a wider range of physical oceanographic conditions and provide a more representative view of dispersal scenarios’. This rationale conflates what is needed to perform appropriate hatchling dispersal simulations (e.g. [37,38]) and what is required to assess the role of ocean currents on a telemetered animal's movement (e.g. [12,39]). In the case of identifying the role of ocean currents on the transport of a specific turtle, ocean conditions that are ‘typical’ or ‘representative’ are not particularly useful as they will tend to average-out the unique oceanic conditions (local weather, tidal phase, etc.) those turtles encountered.

The tracking dataset obtained by Briscoe *et al*. [13] represents a valuable opportunity for research into one of the most important questions in marine ecology. Raising sea turtles for up to 3 years prior to release and developing satellite telemetry attachment methods that lasted between 173 days and 865 days are remarkable achievements. The ocean circulation model (Global HYCOM) and particle tracking software (Ichthyop v. 3.2) employed to estimate ocean velocities are state of the art [28,29]. Given the quality of the datasets that they are working from, robust analyses could be achieved with simple modifications to their present methods.

First, the central aim should be to adequately quantify ocean velocity over the area in which the turtle occurs. To do this, ocean velocities should be obtained along each turtle's track around the area of location uncertainty. If the authors wanted to consider uncertainty in time (as in their initial analyses) or depth, they could release particles from those locations at some time before and after the recorded occurrence and across a range of depths. Ensuring coverage of potentially relevant oceanic conditions must be balanced by the need to compare ocean velocities that were most likely encountered by turtles. Although a number of approaches are possible, a simple way to achieve both goals (and be conservative with respect to concluding that ‘behaviour’ is responsible for differences between track velocity and modelled ocean velocity) is to release a cluster of particles within the spatio-temporal area of uncertainty and select the particle trajectory that best corresponds to the organism's movement to estimate ocean velocity for comparison with track velocity (figure 2). In this way, a wide variety of ocean conditions are accounted for, but only those most closely matching the turtles' movements are used in statistical analyses [40].

For example, a successor to Briscoe *et al*. [13] could release virtual particles along the length of each of the 44 turtles' tracks. Particle velocity estimates could be obtained at daily intervals or, to reduce spatio-temporal autocorrelation (and artificial inflation of sample size for subsequent statistical analyses), some subset of the original tracking data (e.g. every 2, 5, or 10 days). Tracking duration of particles should be set to no more than track duration (particles released at day 1 of a 175 day track would drift for 175 days, particles released at day 2 would drift for 174 days, etc.). The authors could then assess a number of useful metrics including (i) ocean velocity along track segments, (ii) swimming velocity (subtracting ocean velocity from track velocity) along track segments, and (iii) separation distances between particles and the track through time. From these metrics, it would be possible to infer which segments of an individual turtle's track could be accounted for solely by ocean currents and where the turtle likely engaged in oriented swimming [39]. Additionally, this approach would allow for visualization as to whether particle trajectories coincide with the turtle's track. The analyses performed on individual tracks could then be aggregated to gain population-level insight into the movements of turtles [12,40].

Whatever analytical approach is adopted, it should be paired with an identical analysis applied to the tracks of passive oceanographic drifters (figure 3) [4]. Numerous studies show that, over time, trajectories of virtual particles released in ocean circulation models diverge from the tracks of oceanographic drifters [4,24,39,40]. Therefore, before conclusions can be reached about the role of swimming behaviour on the movements of animals, it must first be shown that differences between virtual particles and the animal's track are greater than the differences between virtual particles and the tracks of drifters within the same region. Tracking data for drifters throughout the global ocean are made freely available by the National Oceanographic and Atmospheric Association (NOAA) Atlantic Oceanographic and Meteorological Laboratory (AOML) and can be obtained for specific regions of interest from 1979 to the present (www.aoml.noaa.gov/envids/) [32]. However, comparison with this dataset should be considered the minimum level of scientific rigour, as even the trajectories of drifters deployed in closely spaced pairs can rapidly diverge [12,41]. Ideally, pairs of drifters (or more) would be simultaneously deployed alongside the tracked animals (and drogued at relevant depths) so that separation distances between drifters could be compared with separation distances between turtles and drifters. Moreover, deploying drifters would ensure *in situ* measures of ocean currents were obtained in close proximity to the turtles, at least initially, to test the sensitivity of the ocean circulation model to correctly estimate ocean velocities [12].

## 4. Conclusion

Even small and weakly swimming animals possess a variety of adaptive behaviours that could influence their fate relative to passive drift [6,11,42]. In previous papers, we have argued that natural selection should favour those organisms that bias locomotion in directions that, on average, lead to favourable areas (e.g. [43–45]). As field-based studies become increasingly tractable, the marine ecology community appears primed to expect results that bear-out this paradigm [15,46]. However, given the very few studies in which ocean currents and animal movements have been directly measured, this topic still needs to be carefully considered and scientific standards of acceptance should not be relaxed. As stated in 1968 by F.R. Harden-Jones (p. 224), ‘To determine the relation between the movements of the fish and those of the water, the speed and direction of both must be measured. It is important that the velocity of the current should be measured at the depth at which the fish are swimming. The measurement of current speed and direction raises problems of instrumentation … experiments must be interpreted with care, as the observations may not be accurate enough to resolve the points at issue’ [2]. We encourage future research in this area to employ robust experimental design that uses multiple methods—modelling in the context of *in situ* observations of ocean circulation and organismal movement [11,12]. With careful experimentation and analysis, the extent to which swimming behaviour and ocean currents influence organismal movements can be determined. Such information is sorely needed for enhanced predictions of population-level distributions and, as a result, better conservation and management of marine species, ecosystems, and resources [47].
